# Cell size control is sirtuin(ly) exciting

**DOI:** 10.1038/msb.2013.64

**Published:** 2013-11-12

**Authors:** Jill Wright, Brandt L Schneider

**Affiliations:** 1 Department of Cell Biology and Biochemistry, Texas Tech University Health Sciences Center, Lubbock, TX, USA

In the classic essay ‘On longevity and shortness of life' Aristotle speculated on the causes of the length or brevity of lifespan. More than 23 centuries ago, he postulated that three factors have major effects on longevity: metabolic rate, reproductive rate, and size. Remarkably, these observations still hold true. However, the molecular and genetic mechanisms that are responsible for these functions remain unresolved. In a recent article published in *Molecular Systems Biology*, Moretto et al (2013) used a novel pharmacological and systematic approach to deduce that the yeast sirtuin Sir2 has an integral role in cell size homeostasis. While the role of Sir2 in the regulation of the replicative lifespan of yeast has been firmly established, the proposition by Moretto et al (2013) that Sir2 integrates metabolic signals to modulate both cell size and lifespan is exceptionally provocative.

Cell size homeostasis is a fundamental process that is integrally involved in the regulation of both function and fitness. The biochemical pathways that coordinate cell growth with proliferation to maintain size homeostasis are still being rigorously investigated. Two genome-wide systematic genetic screens for small (*whi*) or large-cell size mutants identified hundreds of genes involved in cell size control (Jorgensen et al, 2002; Zhang et al, 2002). Intriguingly, a recent study by Hoose et al (2012) revealed that the link between cell size and proliferation potential is more complex than previously anticipated.

Building upon previous observations, Moretto et al (2013) confirmed that deletion of *SIR2* increases cell size (Yang et al, 2011). To elucidate a potential role for Sir2 in cell size control, Moretto et al (2013) used the innovative approach of pharmaco-epistasis to systematically examine the impact of inhibiting Sir2 function in combination with gene deletions (*whi*) that reduce cell size. As *sir2* mutants are unable to mate, they used the cleverly conceived idea that a large-scale epistasis analysis could be accomplished by treating individual *whi* mutants with the Sir2 inhibitor nicotinamide, thus effectively creating a *whi sir2*-double mutant.

Moretto et al (2013) first demonstrated that low doses of nicotinamide increase cell size. As nicotinamide failed to increase the size of *sir2* mutants, the observed effects on size were most likely due to the direct inhibition of Sir2. When this approach was applied to 189 *whi* mutants, they found that 22 *whi* mutants remained small, suggesting that these genes likely act downstream of Sir2. Strikingly, 82% of these genes were involved in ribosome biogenesis and nearly 50% were components of the large ribosomal subunit (60S). Subsequent analyses confirmed previous findings that deletions of 60S components markedly reduced cell size (Hoose et al, 2012). Intriguingly, two separate studies revealed that an extensive array of gene deletions for 60S components concomitantly reduced cell size and extended lifespan (Steffen et al, 2008; Yang et al, 2011). Additional experiments suggested a potential direct relationship between cell size and lifespan (Yang et al, 2011; Bilinski et al, 2012). However, the issue is complex and remains contentious (Yang et al, 2011; Bilinski et al, 2012; Ganley et al, 2012; Kaeberlein, 2012).

Elegant work by Steffen et al (2008) demonstrated that diazaborine, a drug that specifically inhibits 60S assembly, extended lifespan and recapitulated the phenotype derived from the loss of large ribosomal subunit components. Building upon this, Moretto et al (2013) demonstrate that diazaborine potently reduces cell size. Moreover, treatment of 155 large-cell size mutants with diazaborine resulted in a subset of 40 mutants, which did not decrease in size, suggesting that these genes function downstream of Sir2. Noteworthy in this group were two genes (*SWI4* and *SWI6*) that encode the SBF transcription factor, which functions as a central node in cell size control (Jorgensen et al, 2002). Furthermore, *swi4* and *swi6* mutants completely blocked the ability of two large ribosomal subunit mutants (*rpl35b* and *rpl37a*) to reduce cell size, suggesting that SBF functions downstream of some aspect of ribosome biogenesis. Extrapolation from these results would suggest that SBF also functions downstream of Sir2. Perhaps even more intriguing is the authors' observation that treatment of cells with nicotinic acid increased the intracellular levels of NAD+, a known activator of Sir2, and reduced the size of wild-type but not *sir2* mutants. Taken together, these results led the authors to speculate that NAD+ is a metabolic regulator of cell size that functions via Sir2 and the 60S ribosomal subunit.

How do these new observations impact our understanding of cell size control? First, the discovery that Sir2 is a potent modulator of cell size is bound to have far-reaching consequences. This discovery adds yet another factor to a myriad of intertwined pathways that are involved in cell size control (Figure 1). On the surface, it appears that this observation is consistent with the hypothesis that increased cell size is somehow linked to decreased lifespan. However, harbingers of discord abound. The authors point out that in some circumstances an increase in cell size coincides with shortened lifespan (e.g., deletion or inhibition of Sir2), while in other cases increased cell size correlates with longevity (e.g., an increase in ploidy). Moreover, even the genetic correlations between cell size and longevity are far from absolute. While many large-cell mutants are short-lived, clearly some are not (e.g., *sir2fob1*). Furthermore, not all small cells are long-lived (e.g., *rpl42b*) nor are all long-lived cells small (e.g., *fob1*). Moretto et al (2013) adroitly point out that the relationship between cell size and aging is intriguing but remarkably complex. As of yet, it appears that there is not a simple link between cell size and lifespan.

The provocative work by Moretto et al (2013) also reinforces current challenges in the field while laying the groundwork and means for addressing new questions (Figure 1). For example, how does Sir2 modulate cell size? The observation that Sir2 modulates both cell size and longevity in yeast is certainly tantalizing. It is widely believed that Sir2 extends lifespan by repressing the formation of extracellular replicating circles (ERCs). Meanwhile, there is circumstantial evidence that increased plasmid load increases cell size (Wang et al, 2009). Perhaps ERCs promote aging by sequestering SBF transcription factors. Regardless, the observations of Moretto et al (2013) further reinforce how intricately entangled are the molecular pathways that modulate cell size with those that regulate longevity.

## Figures and Tables

**Figure 1:**
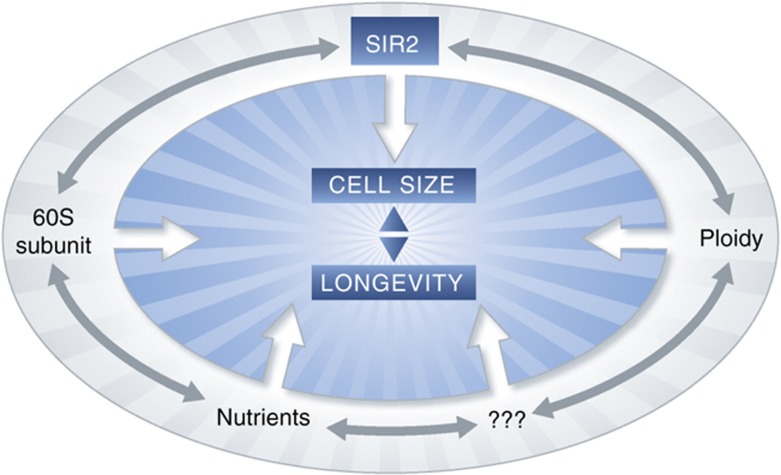
The complexities of cell size control are evident, as multiple both known as well as unknown factors exert effects on size. In addition to affecting cell size directly, these factors also exert effects on one another. Interestingly, in many cases results support an additional, although complex, relationship between cell size and lifespan. However, many questions remain. How does Sir2 modulate cell size? By what mechanisms does the 60S subunit of the ribosome impact cell size? How is it that the small subunit of the ribosome affects cell size in a manner opposite of the large subunit? By what mechanisms do nutrients and ploidy impact cell size and lifespan? Finally, is there a mechanistic relationship between cell size and longevity, and if so, why does increased size not always correlate with a reduced lifespan?
